# Ultrasonic-Assisted Aqueous Two-Phase Extraction and Properties of Water-Soluble Polysaccharides from *Malus hupehensis*

**DOI:** 10.3390/molecules26082213

**Published:** 2021-04-12

**Authors:** Pengcheng Li, Hongkun Xue, Mi Xiao, Jintian Tang, Hansong Yu, Yanqi Su, Xu Cai

**Affiliations:** 1 China Pharmaceutical Preparation Section, Huazhong University of Science and Technology Union Jiangbei Hospital/Wuhan Caidian People’s Hospital, Wuhan 430100, China; LPC951010@163.com (P.L.); xm15926389307@163.com (M.X.); 2 College of Food Science and Engineering, Jilin Agricultural University, Changchun 130033, China; 3 Key Laboratory of Particle & Radiation Imaging, Ministry of Education, Department of Engineering Physics, Tsinghua University, Beijing 100084, China; xuehk@mail.tsinghua.edu.cn (H.X.); 13769140853@163.com (J.T.)

**Keywords:** *Malus hupehensis*, polysaccharide, ultrasonic assisted aqueous two-phase system, structural characterization

## Abstract

*Malus hupehensis* (*M. hupehensis*), an edible and medicinal plant with significant antioxidant and hypoglycemic activity, has been applied to new resource foods. However, the structural characterization and biological effects of its polysaccharides (MHP) are less known. The optimum extraction parameters to achieve the highest extraction efficiency (47.63%), the yield (1.68%) and purity of MHP (89.6%) by ultrasonic-assisted aqueous two-phase system (ATPS) were obtained under the liquid-to-solid ratio of 23 g/mL, ultrasonic power of 65 W, and ultrasonic time of 33 min. According to the analysis results, MHP was composed of Man, GlcA, Rha, GalA, Glc, Gal, Xyl, Ara, and Fuc, in which Ara and Gal were the main components, and the content of GlcA was the lowest. In in vitro activity analysis, MHP showed a significant antioxidant capacity, and an inhibition activity of α-glucosidase and the advanced glycation end products (AGEs) formation in the BSA/Glc reaction model. MHP interacted with α-glucosidase and changed the internal microenvironment of the enzyme, and inhibited the AGEs formation, which provides more evidence for the antihyperglycemic mechanism of MHP. The results suggest that ATPS is an efficient and environmentally friendly solvent system, and *M. hupehensis* has broad application prospects in functional foods, healthcare products, and pharmaceuticals.

## 1. Introduction

*Malus hupehensis* (Pamp.) Rehder (*M. hupehensis*), an edible and medicinal plant, is known as the most important species for ornamental value in the genus *Malus*, and extensively distributed in southern China [1,2]. As an antipyretic drink with time honored history, a pot of tea by using three pieces of leaves is very popular in Hubei province of China [3,4]. It has a variety of pharmacological activities, including antioxidative, antihyperglycemic, antitumor ability, which are established on the presence of polyphenols and flavonoids [5,6]. Owing to its security and health functions, the extract of *M. hupehensis* has been applied to new resource foods and supplements [7,8]. Due to the significant antioxidant and hypoglycemic effect, *M. hupehensis* is a typical traditional Chinese medicine (TCM) and healthy tea to treat type 2 diabetes in the folk traditions [8,9]. However, there is seldom systematic research about polysaccharides from *M. hupehensis* (MHP) contributing to the antioxidant and hypoglycemic effects, which prompted us to perform a detailed investigation.

Polysaccharides, as abundant natural molecules, are widely used for food and pharmaceutical industries. The interesting bioactivities and diverse structures of polysaccharides have been developed in TCM, which makes TCM activate the antioxidant defense system, the immune system, and the endocrine system, etc. [10,11]. For example, antitumor polysaccharides from *Ganoderma lucidum*, three antioxidant polysaccharides obtained from *Agaricus blazei* Murrill, antidiabetic polysaccharides from *Grifola frondosa* Mycelium, four hypoglycemic polysaccharides from corn silk [10,11,12,13]. The extraction and application of polysaccharides is the interesting research point of the medicine and biology field. The structural and biological properties of polysaccharides are closely related to their molecular weight, monosaccharide composition, and three-dimensional structures [14].

The effective extraction of bioactive polysaccharides from natural plants is the significant operation. Various conventional extraction techniques, including hot water extraction, ultrasonic assisted extraction, pressurized water extraction and microwave assisted extraction, etc., have been developed. The efficient and rapid preparation of polysaccharides in one-step is a great challenge [15]. The crude polysaccharides extract should be refined by consuming organic solvents and a complex series of operations prior to applications because of coexisting impurities. However, the repeated elution processes of conventional solid sorben are usually time-consuming, laborious, and lead to the serious loss of target compounds caused by the highly irreversible adsorptive property [16]. The aqueous two-phase system (ATPS) is composed of two kinds of immiscibility polymers or one polymer and salts with water, which have been extensively performed on the extraction and purification of polysaccharides, proteins and other biomolecules in a single step [17]. Polysaccharides were enriched in one phase of APTS under the selectivity of van der Waals’ forces, hydrophobic effect, electrostatic effect, and interfacial tension. Compared with conventional extraction methods, ATPS is the environmentally friendly and highly efficient extraction solvent system that provides a gentle, biocompatible environment for target compounds, and shows the merits of negligible viscosity, quick phase separation, and the excellent solubility [18]. As previously reported, polysaccharides were obtained from aloe leaves, *Ziziphus Jujuba, Solanum nigrum,* and *Lentinus edodes* through ATPS, and these results provided the new possibilities for the rapid extraction and purification of polysaccharides [19,20,21,22]. The extraction efficiency, an important evaluation index of ATPS extraction for polysaccharides, was affected by many factors, which needs to be further elucidated.

In this study, the objective of this work was to (1) optimize the extraction conditions for MHP by the response surface methodology (RSM), then establish a rapid and effective extraction method based on ultrasonic-assisted aqueous two-phase system (UAATPS); (2) analyze the preliminary characterizations of MHP according to chemical analysis and modern chromatography; (3) study the potential antioxidant and hypoglycemic activity of MHP for food and pharmaceutical applications.

## 2. Results and Discussion

### 2.1. Selection of Ethanol/Salt ATPS

**Figure 1:**
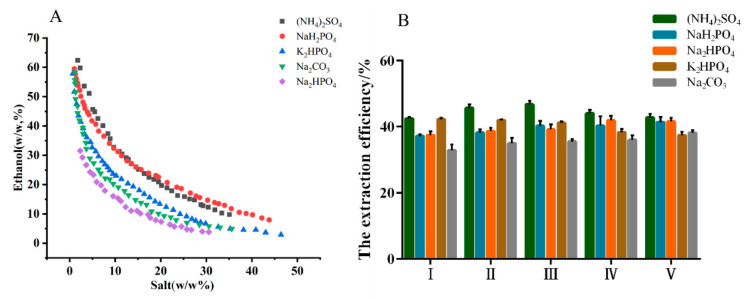
Binodal curves for aqueous two-phase system composed of ethanol/salts at 25 °C, and atmospheric pressure (**A**), and the effect of the compositions of aqueous two-phase system on the extraction efficiency of polysaccharides from *Malus hupehensis* (**B**): mass fraction of ethanol/salt (%).

The phase diagrams and extraction efficiency of different ATPSs are shown in Figure 1. The phase-separation ability of five salts were ranked in the order as follows: (NH_4_)_2_SO_4_ < NaH_2_PO_4_ < K_2_HPO_4_ < Na_2_CO_3_ < Na_2_HPO_4_. The order was consistent with the phase forming ability of different salts that are quantitatively described by Gibbs free energy of hydration [23,24]. However, the extraction efficiency of MHP was inversely proportional to the phase-separation ability. (NH_4_)_2_SO_4_ was more beneficial as the phase-forming salt based on the higher extraction efficiency. In addition, when the concentration of (NH_4_)_2_SO_4_ was located in the range of 18–23% (*w/w*), the extraction efficiency of MHP improved with the increase in (NH4)_2_SO_4_ concentration.

A maximum extraction efficiency of 46.70% was obtained at the (NH4)_2_SO_4_ concentration of 19.86% (*w/w*), which may be attributed to the salt-out effect. Therefore, ethanol/(NH4)_2_SO_4_ ATPS was selected as the extraction system for the following studies, and the optimal parameters were as follows: ATPS composition of ethanol concentration at 30.31% (*w/w*) and (NH4)_2_SO_4_ concentration at 19.86% (*w/w*).

### 2.2. RSM Optimization

#### 2.2.1. Single Factor Experiments

**Figure 2:**
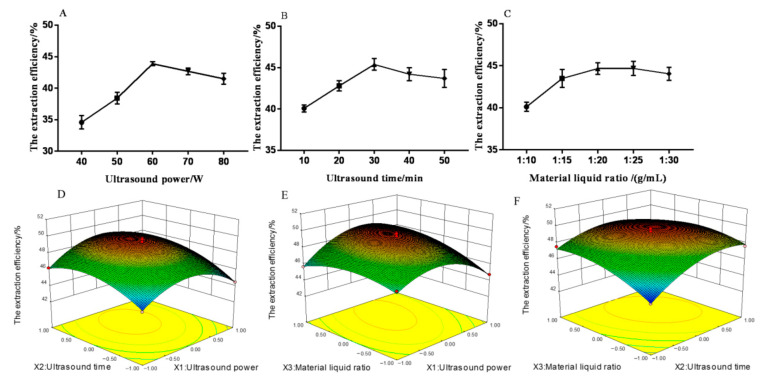
Effect of ultrasonic power (**A**), ultrasonic time (**B**), material–liquid ratio (**C**), ultrasonic power and ultrasonic time (**D**), ultrasonic power and material–liquid ratio (**E**), ultrasonic time and material–liquid ratio (**F**) on the extraction efficiency of polysaccharides from *Malus hupehensis*.

The ultrasonic time (10–50 min) and material–liquid ratio (1:10–1:30 g/mL) on the extraction efficiency of MHP were studied to optimize the extraction process and determine the influence of ultrasonic power (40–80 W). The results of single factor experiments showed that the extraction efficiency of MHP was correlated with ultrasonic power, ultrasonic time, and material–liquid ratio. As shown in Figure 2A, at a certain range, the extraction efficiency of MHP could be increased by increasing the ultrasonic power. The increase in ultrasonic power could promote the fragmentation of plant tissue and improve the solubility of MHP. The maximum extraction efficiency of MHP was 43.98% when the ultrasonic power was 60 W. The extraction efficiency of MHP decreased with the ultrasonic power exceeding 60 W because the structure of MHP was changed and degraded due to the high ultrasonic power [25]. As shown in Figure 2B, at a certain range, the extraction efficiency of MHP could be increased by increasing the ultrasonic time. The longer the extraction time, the stronger the heat accumulation effect, thus promoting the diffusion rate of MHP. The extraction efficiency of MHP reached a maximum value of 45.31% at 30 min. The increase in ultrasonic time could destroy the cell tissue and dissolve the MHP solute. When the ultrasonic time was further increased, the extraction efficiency of MHP decreased due to the structural change and degradation of MHP because of the long ultrasonic time and the enhanced thermal accumulation effect [26]. Figure 2C shows that when the material–liquid ratio was 1:10–1:20 (g/mL), the extraction efficiency of MHP increased significantly with the increase of this ratio (*p* < 0.05), and the maximum extraction efficiency of MHP reached 45.39% when the ratio was 1:20 (g/mL). The reason was when the material–liquid ratio was increased, the concentration difference between the internal and external solvents of the plant tissue was larger and the permeability and diffusion force of MHP increased, thus promoting the dissolution of MHP solute. When the material–liquid ratio was further increased, the extraction efficiency of MHP decreased due to the decrease in concentration gradient between the solute and the solvent and the decrease in MHP diffusion force [27]. Meanwhile, the loss of MHP increased and the extraction efficiency of MHP decreased slowly in the process of MHP collection. Therefore, three factors and three levels were selected for response surface test: ultrasonic power of 50–70 W, ultrasonic time of 20–40 min, and material–liquid ratio of 1:15–1:25 g/mL.

#### 2.2.2. Model Establishment and Significance Test

The optimal process conditions of response surface were optimized in accordance with the results of single factor experiments. The experimental design and results are shown in Table S4. The extraction efficiency of MHP (*E*) used as the response value, the regression Equation (1) of ultrasonic power *X*_1_, ultrasonic time *X*_2_, and material–liquid ratio *X*_3_ with the extraction efficiency were obtained.

(1) $$\begin{array}{l} E=49.32+0.11X_{1}+0.97X_{2}+0.68X_{3}+0.34X_{1}X_{2}\\ +0.89X_{1}X_{3}-0.67X_{2}X_{3}-2.23X_{1}^{2}-1.44X_{2}^{2}-1.01X_{3}^{2} \end{array}$$

Table S4 shows the results of ANOVA. *F* value could test the significance of each variable on the response value. The larger the *F* value, the higher the significance of the corresponding variables. The model significance test was *p* < 0.05, and the determination coefficient (*R*^2^) was 0.9727. The model showed a statistical significance, but the lack-of-fit was not significant. Therefore, the response model could be used to analyze and predict the optimal extraction process of MHP.

#### 2.2.3. Interaction Analysis

In accordance with the regression equation, a three-dimensional surface graph was established to determine the optimal extraction conditions. The results are shown in Figure 2. The interaction between ultrasonic power and material–liquid ratio as well as ultrasonic time and material–liquid ratio significantly affected the extraction efficiency of MHP (*p* < 0.05). The interaction of other factors had no significant effect on the extraction efficiency of MHP.

The regression equation was further analyzed by Design-Expert software, and the optimal process conditions were obtained as follows: ultrasonic power of 65 W, ultrasonic time of 33 min, material–liquid ratio of 1:23 g/mL, and the theoretical extraction efficiency of MHP of 48.78%. Validation test was carried out to verify the reliability of the method, the average value was 47.63% after the test was repeated three times. The MHP yield was 1.68%, and the purity was 89.6%. The results showed that the model could well simulate and predict the content of MHP, thereby proving the feasibility of the REM optimization process.

### 2.3. FT-IR Analysis

**Figure 3:**
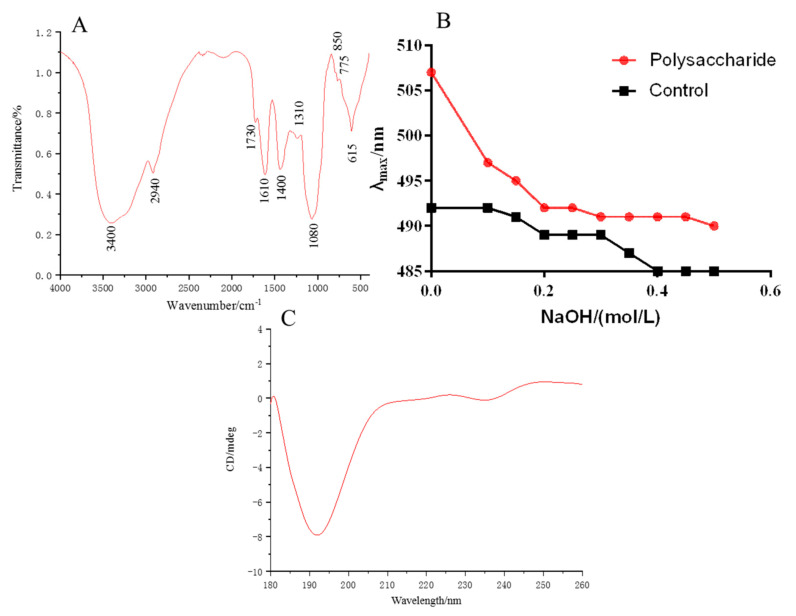
FT-IR spectra of polysaccharides (**A**), maximum absorption wavelength of polysaccharides-Congo red (**B**) and CD chromatogram (**C**).

The characteristic absorption peaks of MHP are shown in Figure 3A. The O–H stretching vibration at 3400 cm^−1^ and the C–H stretching vibration at 2940 cm^−1^ were the characteristic absorption peaks of polysaccharide [28]. Absorption peaks at 1730 and 1420 cm^−1^, which were asymmetric COO stretching vibration, were also found. The peaks indicated that galacturonic acid may exist in MHP. The symmetrical COO stretching vibration at 1610 cm^−1^ indicated the existence of ester group in MHP [29]. The stretching vibration of C–O with ether bond at 1080 cm^−1^ belonged to the asymmetric absorption peak of glycosidic bond, and the weak absorption peak at 845 cm^−1^ indicated a small amount of α-glycosidic bond [28].

### 2.4. Monosaccharide Composition Analysis

The chromatogram of PMP derivatization of MHP hydrolysate is shown in Table 1. MHP was composed of Man, GlcA, Rha, GalA, Glc, Gal, Xyl, Ara, and Fuc, with molar ratios of 3.22:1.85:4.25:5.08:6.69:38.46:4.68:30.90:4.87, and the contents of Gal and Ara were the highest at 38.46% and 30.90%, respectively. The results showed that MHP was mainly composed of Gal and Ara. Some studies have shown that polysaccharides with Ara showed a certain hypoglycemic effect. For example, when starch was eaten with Ara, it could effectively reduce the blood sugar level; when Ara instead of sucrose, it may reduce blood glucose; Ara can inhibit the absorption of sucrose and alleviate high glucose induced oxidative stress [30,31]. Therefore, MHP may have a good hypoglycemic effect.

### 2.5. Congo Red and Circular Dichroism Analysis

Congo red could detect whether polysaccharides have a triple helix structure. The polysaccharide with triple helix structure could form a complex with Congo red, and its maximum absorption wavelength increases and red shift occurs. Figure 3B illustrates that when the NaOH concentration increased, the maximum absorption wavelength of the solution decreased, indicating that the triple helix structure of MHP was destroyed by the higher concentration of NaOH and could not form a complex with Congo red. Thus, its red shift disappeared. As shown in Figure 3C, an obvious negative button effect existed at 193 nm, indicating that MHP has an asymmetric structure.

### 2.6. In Vitro Antioxidant Activity

**Figure 4:**
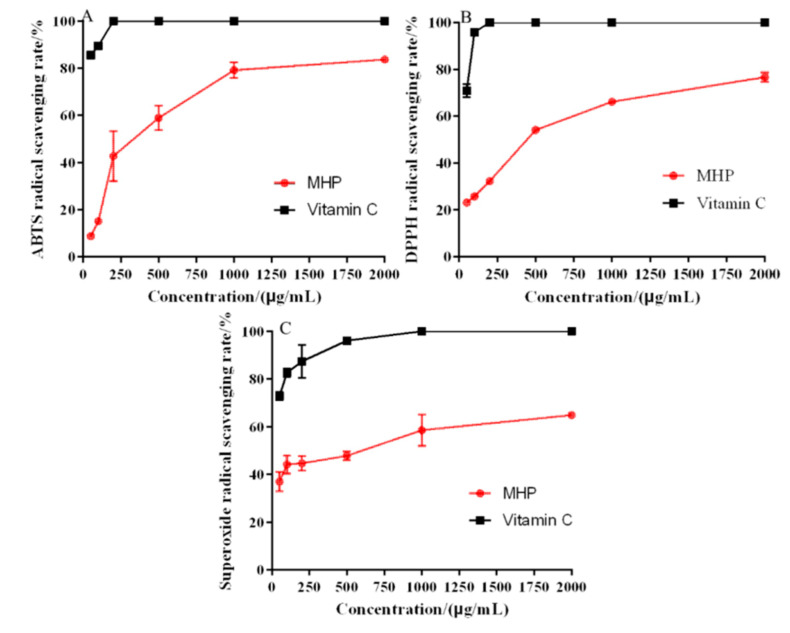
In vitro antioxidant activity: the scavenging rate of ABTS (**A**), DPPH (**B**), and superoxide radical (**C**).

The antioxidant activity of MHP was determined by DPPH, ABTS, and hydroxyl and superoxide radical systems, and vitamin C was used as the positive control. ABTS radical scavenging capacity detection method could be used to detect the antioxidant capacity of hydrophilic and lipophilic substance, and it is the most widely used indirect detection method [32]. The results of ABTS radical scavenging activity of MHP are shown in Figure 4A. The ability of ABTS radical scavenging increased with the increase in concentration. When the MHP concentration was 2000 μg/mL, the strongest scavenging effect of MHP on ABTS free radical was 57.59% ± 3.67% and the IC_50_ value was 467.49 ± 13.72 μg/mL.

DPPH radical scavenging mechanism was based on DPPH radical as hydrogen ion acceptor; thus, DPPH was converted into a non-free radical (DPPH-H) [33]. Figure 4B shows that the DPPH radical scavenging ability of MHP was related to its concentration. In the range of 50–2000 μg/mL, the scavenging capacity was significantly enhanced, and the maximum scavenging rate was 75.42% ± 1.26%. In general, the lower the IC_50_ value, the stronger the antioxidant activity. The IC_50_ value of DPPH radical scavenging activity was 487.57 ± 8.67 μg/mL. The results showed that MHP could be used as a hydrogen donor to combine with DPPH radical to form a more stable substance, thus scavenging DPPH radicals.

Superoxide free radical is a kind of reactive oxygen free radical produced in the human body. It could trigger lipid peroxidation in the body. Therefore, the antioxidant activity of compounds could be evaluated by scavenging superoxide radicals in vitro [34]. As shown in Figure 4C, the superoxide radical scavenging was the strongest at 2000 μg/mL, which was 64.87% ± 2.63%, and the IC_50_ value was 684.53 ± 8.39 μg/mL.

The antioxidant activity of MHP extracted using UAATPS showed a dose-dependent relationship as its antioxidant capacity increased with the increase in MHP concentration. Compared with polysaccharides from wheat bran, dandelion roots and *Porphyra haitanensis*, MHP has a significant antioxidant activity [33,34,35]. It is therefore a source of active compounds that might be used as potential antioxidant.

### 2.7. In Vitro Antihyperglycemic Activity

#### 2.7.1. α-Glucosidase Inhibitory Activity

**Figure 5:**
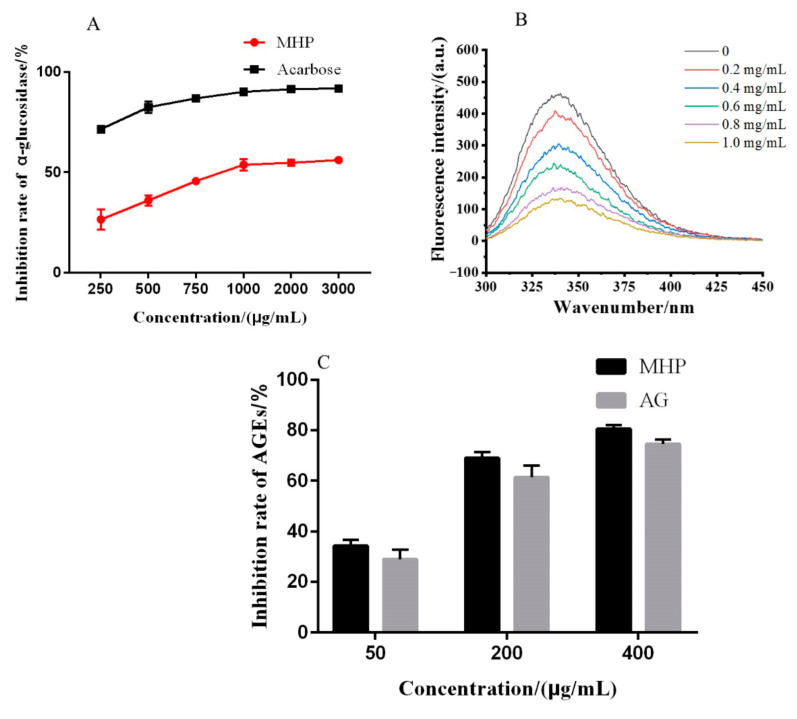
α-glucose inhibitory activity (**A**), the quenching effect of polysaccharides on fluorescence spectra of α-glucosidase (**B**) and effect of polysaccharides on the formation of fluorescent AGEs (**C**).

α-glucosidase inhibitors could inhibit enzyme activity and slow down the decomposition of sugars in the human body, thereby delaying the absorption rate of glucose. Therefore, they play an important role in controlling blood glucose and diabetes. As shown in Figure 5A, the inhibitory effect of MHP on α-glucosidase showed a concentration-dependent relationship at the concentration of 250–3000 μg/mL, and its IC_50_ value was 994.52 ± 10.63 μg/mL. Although the inhibitory activity of MHP is worse than that of acarbose and wheat bran polysaccharides, as a natural product, it does not produce side effects on the human body [36,37]. Thus, it shows a great hypoglycemic potential.

According to the amino acid sequence of α-glucosidase from *Saccharomyces cerevisiae* with NCBI, α-glucosidase is composed of 589 amino acid residues and 20 tryptophan (Trp) residues, which could provide intrinsic fluorescence. When a fluorescent substance interacts with other substances, its fluorescence intensity decreases, which is called fluorescence quenching. The fluorescence intensity of α-glucosidase gradually weakened with the increase MHP concentration. Results indicated that the fluorescence of α-glucosidase was affected by concentration-dependent quenching. Moreover, the absorption peak of α-glucosidase (about 331 nm) gradually shifted to the right under different MHP concentrations (Figure 5B). The right shift phenomenon implied that the polarity of the microenvironment around Trp residues in α-glucosidase changed and became more hydrophilic after the addition of MHP [38]. Many studies showed that the polysaccharide with moderate molecular weight and high water solubility has the biological activity, while that with double helix or triple helix structure has better biological activity than other polysaccharides [14]. In the present study, MHP had a certain triple helix structure. Therefore, the α-glucosidase inhibitory activity was related to the change of the microenvironment around Trp residues in α-glucosidase that may be caused by the triple helix structure of MHP.

#### 2.7.2. Inhibition of MHP on AGEs Formation

Studies have shown that continuous hyperglycemia causes nonenzymatic glycosylation of many proteins and the formation of AGEs, which play an important role in the pathogenesis of chronic complications of diabetes, senility, kidney dysfunction, and cardiovascular disease [39]. In the present study, the capability of antiglycation of MHP was evaluated on the basis of the inhibition rate of fluorescent AGEs. As shown in Figure 5C, the inhibitory effect of AGEs was dependent on the concentration of MHP. At a concentration of 400 μg/mL, the inhibition ratio of MHP reached around 80%, and the IC_50_ value was 143.78 ± 5.62 μg/mL. It was reported that polysaccharides from pumpkin and *Coptis chinensis* showed the similar antiglycation effect [39,40]. Therefore, MHP can be used to decrease AGEs in human body to help prevent or control diseases like diabetes and senility.

## 3. Materials and Methods

### 3.1. Material and Chemicals

*Malus hupehensis* (Pamp.) Rehder was purchased from Lishizhen Chinese herbal medicine market (Huanggang, Hubei Province, China). The samples were identified by Professor Bisheng Huang, Department of Pharmacy, Hubei University of Chinese Medicine. Specimens of the samples were deposited in Department of Engineering Physics, Tsinghua University (Voucher Specimen Number, 20201028). The samples were smashed and sieved by an 80 meshes sieve, extracted by petroleum ether for 2 h, then dried at 45 °C for the extraction experiment.

Arabinose (Ara), galactose (Gal), glucose (Glc), fructose (Fru), xylan (Xyl), rhamnose (Rha), mannose (Man), glucuronic acid (GlcA), galacturonic acid (GalA), trifluoroacetic acid (TFA), acarbose, porcine pancreatic *α*-amylase, *α*-glucosidase, p-nitrophenyl-α-D-glucopyranoside (pNPG), bovine serum albumin (BSA), 1,1-diphenyl-2-picric acid (DPPH), 2,2′- diazo-bis-3-ethylbenzothiazoline-6-sulfonic acid (ABTS), Folin ciocalteu reagent were purchased from Shanghai Huicheng Biotechnology Co., Ltd. (Shanghai, China); Na_2_CO_3_, (NH_4_)_2_SO_4_, K_2_HPO_4_, Na_2_HPO_4_, NaH_2_PO_4_, HCl, ethanol, and Congo red were analytical grade, and purchased from Merck Chemical Technology (Shanghai) Co., Ltd. (Shanghai, China). Methanol (Thermo Fisher Scientific, Waltham, MA, USA) was HPLC grade in HPLC experiment.

### 3.2. Preparation of ATPS

As the turbidity titration method previously described [41], the ATPS of ethanol/salt system and the phase diagrams of ATPS were carried out as follows: salt (Na_2_CO_3_, (NH_4_)_2_SO_4_, K_2_HPO_4_, Na_2_HPO_4_, NaH_2_PO_4_), water and ethanol were mixed well and cultivated at 50 °C. With the addition of salt and water, the weight and volume ratios of salt and ethanol were recorded at the phase transformation point that the system showed two phase separation, respectively. Finally, the phase diagrams of ATPS were plotted on the basis of the recorded data.

### 3.3. Extraction of MHP

The samples were added into the prepared ATPS (Table S1) at the material–liquid ratio of 1:20 g/mL, then sonicated in an ultrasonic bath (UP-250, Shanghai Bilang Instrument Manufacturing Co., Ltd. China) at an ultrasound power of 50 W for 20 min. The extraction temperature was set to 50 °C. The upper and lower phases were collected and filtrated, respectively. The solution was dialyzed in distilled water for 3 days (3500 Da, Beijing Ruida Henghui Technology Development Co., Ltd., Beijing, China), then a vacuum freeze drier was used to obtain MHP. Lastly, the extraction efficiency (*E*), the extraction yield (*Y_L_*) and the purity (*P_L_*) of MHP were analyzed by phenol sulfuric acid method with glucose standard, and shown in Equations (2)–(4):

(2) $$Y_{L}(\%)=\frac{C_{L}V_{L}}{M_{1}}\times 100$$

(3) $$P_{L}(\%)=\frac{C_{L}V_{L}}{M_{2}}\times 100$$

(4) $$E(\%)=\frac{C_{L}V_{L}}{C_{L}V_{L}+C_{U}V_{U}}\times 100$$

where *V_L_*, *C_L_* are the volume of lower phase (mL) and the concentration of MHP (μg/mL), respectively. *V_U_*, *C_U_* are the volume of upper phase (mL) and the concentration of MHP (μg/mL), respectively. *M*_1_, *M*_2_ are the quality of the *M. hupehensis* powder and the dry matter of lower phase (mg).

### 3.4. Optimization of Extraction Condition

A suitable range of experimental factors, including material–liquid ratio (*X*_1_, 10–30 mL/g), ultrasonic power (*X*_2_, 40–80 W), ultrasonic time (*X*_3_, 10–50 min), were selected to the improvement of the extraction efficiency, and carried out by a single-factor design. In accordance with the single-factor experimental results, a three-level-three-variable Box-Behnken design (BBD) was conducted to optimize the best combination of extraction variables (*X*_1_, *X*_2_, *X*_3_) at three levels (Tables S2 and S3). The extraction efficiency of MHP (*E*) was used as the response value. The relationship between the independent and dependent variables is shown in Equation (5):

(5) $$E=\beta_{0}+\sum_{j=1}^{k}\beta_{j}X_{j}+\sum_{j=1}^{k}\beta_{jj}X_{j}^{2}+\sum_{i}\sum_{<j=2}^{k}\beta_{ij}X_{i}X_{j}+e_{i}$$

where *E* is the extraction efficiency of MHP (%); *X_i_* and *X_j_* are the coded variables (*i* and *j* range from 1 to *k*); *β*_0_, *β_j_*, *β_jj_*, and *β_ij_* are regression coefficients of intercept coefficient, linear, quadratic, and the second-order terms, respectively; *k* is the number of independent parameters (*k* = 4) and *e_i_* is the error. The applicability of the predicted polynomial model and the optimized extraction conditions was evaluated by the coefficients of determination (*R*^2^), F-test (*p* = 0.05), and an additional experiment, respectively.

### 3.5. Characterization of MHP

#### 3.5.1. Flourier Transformation Infrared Spectroscopy (FT-IR) Analysis

According to the ratio of 1:100, MHP (2.0 mg) was mixed with KBr, and the mixture was grinded and pressed together. The wavelength of FTIR Spectrometer (Shanghai Cupboard technology development Co., Ltd., Shanghai, China) was set at 4000–400cm^−1^.

#### 3.5.2. Congo Red Test

MHP solution (1.0 mL, 2.0 mg/mL) and Congo red reagent (1.0 mL, 80.0 μmol/L) were mixed thoroughly. Then NaOH solution (1.0 mol/L) was gradually added to obtain different concentrations of NaOH solution (0, 0.1, 0.15, 0.2, 0.25, 0.3, 0.35, 0.4, 0.45, 0.5 mol/L). Water was set as the control group. Finally, the maximum absorbance of NaOH solution was measured by UV spectrometer (Beijing Planck New Technology Co., Ltd., Beijing, China), and the curves of concentration–maximum absorption wavelength were obtained.

#### 3.5.3. Circular Dichroism (CD) Spectroscopy Analysis

The asymmetric structure and structure change of WHP was determined by CD spectrum. WHP solution (3 mg/mL) was used in this study. The spectra were recorded in the far UV range (200–250 nm) with a 1 mm quartz cell on a J-810 CD spectrophotometer (JASCO Co., Tokyo, Japan). The setting conditions of CD spectra are as follows: the scanning rate of 60 nm/min, spectral resolution of 1 nm, response of 1 s, and bandwidth of 1 nm.

#### 3.5.4. Monosaccharide Composition Analysis

The monosaccharide composition and mole ratio of MHP were quantitatively analyzed by HPLC combined with precolumn derivatization method. MHP (5 mg) were dispersed in TFA (2 mL, 4 M) sealed tube, and hydrolyzed to monosaccharides at 110 °C. Then, the sample solution was derived by using PMP-HPLC analysis. Finally, the derivatization product of MHP was filtered, and stored for the following HPLC analysis.

The HPLC equipment was an ultimate 3000 system (Thermo Scientific, USA) in the isocratic elution mode. The experiment conditions were set as follows: COSMOSIL 5C_18_-PAQ 4.6 × 250mm was eluted with acetonitrile and phosphate buffer as mobile phase at a flow rate of 1.0 mL/min at 35 °C.

### 3.6. Assay for Antioxidant Activity of MHP

The DPPH, ABTS and superoxide radical scavenging activities were evaluated according to the method previously described with some modifications [32]. Ascorbic acid was used as a positive control. The radical scavenging was calculated according to the following Equation (6):

(6) $$Scavenging\;activity(\%)=\left(1-\frac{A_{i}-A_{j}}{Ao}\right)\times 100$$

where *A_i_*, *A*_0_, *A_j_* was the absorbance of sample, blank control (without sample), the blank reagent, respectively. Sample concentration providing 50% inhibition (IC_50_) was calculated according to the graph plotting inhibition percentage.

### 3.7. In Vitro Antihyperglycemic Activity

#### 3.7.1. α-Glucosidase Inhibitory Activity

The antihyperglycemic activity of MHP in vitro was evaluated by α-glucosidase inhibition ability according to the method, that was slightly modified [38]. In short, 50 μL α-glucosidase (0.5 U/mL, dissolved in 0.2 M PBS, pH 6.9) was mixed with 50 μL MHP sample solution of different concentrations (0.2–3.0 mg/mL), and then incubated at 37 °C for 10 min. Then, 50 μL pNPG (5 mM) was added and incubated at 37 °C for 20 min. After that, 80 μL of Na_2_CO_3_ solution (0.2 M) was added to stop the reaction and the absorbance was detected at 405 nm. Acarbos was used as the positive control and blank control without enzyme. The α-glucosidase inhibitory activity was calculated according to the following Equation (7):

(7) $$Inhibition\;rate(\%)=\left(1-\frac{A_{s}-A_{b}}{A_{o}}\right)\times 100$$

Among them, *A_s_*, *A_b_*, and *A*_0_ are the absorbance of the test sample system, the mixture of pNPG and MHP without enzyme, and the mixture of pNPG and enzyme without MHP, respectively.

Fluorescence measurements were preformed via a DSF-150WT fluorescence spectrometer (Disofoo measurement technology Co., Ltd., Jiangsu, China). The final concentration of α-glucosidase was prepared to be 1 U/mL in all solutions, while the different MHP final concentrations in the solutions were 0, 0.2, 0.4, 0.6, 0.8, 1 mg/mL, respectively. α-glucosidase and MHP were mixed well. The excitation wavelength and excitation slit were 280 and 5 nm, respectively. The fluorescence spectra of each mixture was obtained in the range of 300-450 nm [19,20].

#### 3.7.2. Inhibition of MHP on Advanced Glycation end Products (AGEs) Formation

To study the capability of antiglycation of MHP, BSA/Glc and aminoguanidine were used as the reaction model and positive control, respectively [42]. A series of MHP (50, 200 and 400 μg/mL) were added into the mixed PBS solution (0.1 M, pH 7.4) of BSA (240 μL, 20 mg/mL) and Glc (120 μL, 20 mmol/L), then incubated together in a capped test tube (10 mL) at 80 °C. After 24 h, the content of fluorescent AGEs was determined by a DSF-150WT fluorescence spectrometer at the excitation wavelength 360 nm, the emission wavelength 453 nm. The inhibition of MHP on AGEs was calculated according to the following Equation (8):

(8) $$Inhibition\;rate(\%)=\frac{F-F_{0}}{F_{0}}\times 100$$

where *F, F*_0_ was the fluorescence intensity of sample, blank control (without sample), respectively.

## 4. Conclusions

*Malus hupehensis* (*M. hupehensis*), an edible and medicinal plant with significant antioxidant and hypoglycemic activity, has been applied to new resource foods. However, the structural characterization and biological effects of its polysaccharides (MHP) are less known. The optimum extraction parameters to achieve the highest extraction efficiency (47.63%), the yield (1.68%) and purity of MHP (89.6%) by ultrasonic-assisted aqueous two-phase extraction (ATPS) were obtained under the liquid-to-solid ratio of 23 g/mL, ultrasonic power of 65 W, and ultrasonic time of 33 min. The physicochemical properties and biological activity of MHP were investigated. According to the analysis results, MHP was composed of nine monosaccharides including Man, GlcA, Rha, GalA, Glc, Gal, Xyl, Ara, and Fuc, in which Ara and Gal were the main components, and the content of GlcA was the lowest. In in vitro activity analysis, MHP showed a significant antioxidant capacity, and an inhibition activity of α-glucosidase and the AGEs formation in the BSA/Glc reaction model. The biological properties of polysaccharides might be attributed to the physicochemical characteristic of MHP. According to fluorescence analyses, MHP interacted with α-glucosidase and changed the internal microenvironment of the enzyme, and inhibited the AGEs formation, which provided more evidence for the antihyperglycemic mechanism of MHP. The results suggest that ATPS is an efficient and environmentally friendly solvent system, and *M. hupehensis* has broad application prospects in functional foods, healthcare products, and pharmaceuticals.

## Figures and Tables

**Table 1:** Monosaccharide composition analysis.

Monosaccharide Composition (%)								
Man	GlcA	Rha	GalA	Glc	Gal	Xyl	Ara	Fuc
3.22	1.85	4.25	5.08	6.69	38.46	4.68	30.90	4.87

## Data Availability

Research data are not shared.

## Supplementary Materials

The following are available online, Table S1: Experimental design and results of response surface methodology: Ethanol/salt ratio of ATPS, Table S2: Experimental design independent variables and their levels, Table S3: Experimental design and results of response surface methodology.

## Sample Availability

Samples of *Malus hupehensis* and polysaccharides are available from the authors.
