# Supplementary material for: Ultrasonic-Assisted Aqueous Two-Phase Extraction and Properties of Water-Soluble Polysaccharides from Malus hupehensis
Source: Molecules. 2021 Apr 12;26(8):2213. doi: 10.3390/molecules26082213 (PMC8068786; doi:10.3390/molecules26082213)
Supplement: Supplementary file 1 [file molecules-26-02213-s001.pdf]

Electronic Supplementary Material

Ultrasonic-assisted aqueous two-phase extraction and properties of water-soluble polysaccharides from *Malus hupehensis*

Pengcheng Li[1,2#], Hongkun Xue[3#], Mi Xiao[1], Jintian Tang[3], Yanqi Su[1*], Hansong Yu[2*], Xu Cai[3*]

[1]China Pharmaceutical Preparation Section, Huazhong University of Science and Technology Union Jiangbei Hospital/Wuhan Caidian People's Hospital, Wuhan 430100, P.R.China

[2]College of Food Science and Engineering, Jilin Agricultural University, Changchun 130033, China

[3]Key Laboratory of Particle & Radiation Imaging, Ministry of Education, Department of Engineering Physics,Tsinghua University, Beijing 100084, P.R.China

[#]These authors contributed equally.

*Correspondence: E-mail: caixu@mail.tsinghua.edu.cn; yuhansong@jlau.edu.cn; suyanqi@sohu.com; Tel: +86-010-627967

Table S1. Ethanol/salt ratio of ATPS

NO.	Ethanol/salt ratio (w/w, %)
I	28.43/22.36
II	29.84/20.78
III	30.31/19.86
IV	31.74/18.25
V	31.74/18.25

Table S2. Experimental design independent variables and their levels

Levels	Independent Variables		
	**material-liquid ratio (X_1, mL/g)**	**ultrasonic power (X_2, W)**	**Extraction time (X_3, min)**
−1	15	50	20
0	20	60	30
1	25	70	40

Table S3. Experimental design and results of response surface methodology

NO.	Variables			The extraction efficiency (%)
	X_1 (mL/g)	X_2 (%)	X_3 (ºC)	
1	-1	-1	0	46.43
2	1	-1	0	45.67
3	-1	1	0	44.71
4	1	1	0	47.53
5	-1	0	-1	47.58
6	1	0	-1	47.51
7	-1	0	1	47.58
8	1	0	1	47.92
9	0	-1	-1	44.74
10	0	1	-1	44.45
11	0	-1	1	46.19
12	0	1	1	47.25
13	0	0	0	49.76
14	0	0	0	49.45
15	0	0	0	49.65
16	0	0	0	48.39
17	0	0	0	49.34

Table S4. ANOVA of response surface models for the extraction efficiency of polysaccharides

Source	Squares	df	Square	Value	Prob > F	significant
Model	53.98	9	6.00	27.69	0.0001	*
A	0.10	1	0.10	0.48	0.5116	
B	7.51	1	7.51	34.67	0.0006	*
C	3.67	1	3.67	16.96	0.0045	*
AB	0.46	1	0.46	2.10	0.1902	
AC	3.20	1	3.20	14.80	0.0063	*
BC	1.80	1	1.80	8.29	0.0237	*
A^2	20.85	1	20.85	96.28	< 0.0001	*
B^2	8.67	1	8.67	40.05	0.0004	*
C^2	4.28	1	4.28	19.75	0.0030	*
Residual	1.52	7	0.22			
Lack of Fit	0.33	3	0.11	0.37	0.7782	
Pure Error	1.18	4	0.30			
Cor Total	55.49	16				
	R^2=0.9727		$C.V.$=0.99	Adj R^2 =0.9376		
